# Miscellany

**Published:** 1891-02

**Authors:** 


### Miscellany.

THE MATTISON PRIZE. OPIUM ADDICTION AS RELATED TO RENAL DISEASE. A PRIZE OF FOUR HUNDRED DOLLARS. With the object of advancing scientific study and settling a now mooted question, Dr. J. B. Mattison, of Brooklyn, offers a prize of $400 for the best paper on Opium Addiction as Related to Renal Disease, based upon these queries:

Will the habitual use of opium, in any form, produce organic renal disease?

If so, what lesion is most likely?

What is the rationale?

The contest is to be open for two years from December 1, 1890, to either sex, and any school or language.

The prize paper is to belong to the American Association for the Cure of Inebriety, and be published in a New York medical journal, *Brooklyn Medical Journal*, and *Journal of Inebriety*.

Other papers presented are to be published in some leading medical journal, as their authors may select.

All papers are to be in possession of the Chairman of Award Committee on or before January 1, 1893.

The Committee of Award will consist of Dr. Alfred L. Loomis, President New York Academy of Medicine, Chairman; Drs. H. F.

Formad, Philadelphia; Ezra H. Wilson, Brooklyn; Geo. F. Shrady, and Jos. H. Raymond, editor *Brooklyn Medical Journal.*

---

Civil Service Examination.—An open competitive examination of candidates for superintendent and first assistant physician in any of the state hospitals and asylums, will be held at the rooms of the Civil Service Commission, Albany, N. Y., Thursday, March 5, 1891, commencing at 10 o'clock, A. M..

A candidate for the position of superintendent must be a citizen of the State of New York, at least thirty years of age, and have had at least five years' actual experience as a physician in a hospital for the insane.

A candidate for the position of first assistant physician must be a citizen of the State of New York, twenty-five years of age, and have had at least three years' actual experience as a physician in a hospital for the insane.

Also at the same time and place, candidates for female physician, as provided in Chapter 243, Laws of 1890, will be examined.

Application blanks may be had by addressing the secretary of the New York Civil Service Commission, Albany, N. Y.

John B. Riley, Chief Examiner.

Albany, N. Y., January 14, 1891.

---

Announcement.—E. B. Treat, publisher, N. Y., has in press for early publication the Ninth Yearly issue of the *International Medical Annual.* Its corps of thirty-seven editors—specialists in their respective departments, comprising the brightest and best American, English and French authors—will vie with previous issues in making it even more popular and of more practical value to the medical profession. We have the assurance of some of the best medical practitioners, that the service rendered their profession by this *Annual* cannot be duplicated by any current annual or magazine, and that it is an absolute necessity to every physician who would keep abreast with the continuous progress of practical medical knowledge. Its index of new remedies and dictionary of new treatment, epitomized in one ready reference volume, at the low price of $2.75, make it a desirable investment for the busy practitioner, student and chemist.

